# Autologous bone marrow transplanation for extramedullary plasmacytoma presenting as adrenal incidentaloma

**DOI:** 10.4103/0256-4947.51785

**Published:** 2009

**Authors:** Mohammed Ahmed, Abduallah Al-Ghamdi, Mohammed Al-Omari, Mahmoud Aljurf, Yusuf Al-Kadhi

**Affiliations:** a From the Department of Medicine, King Faisal Specialist Hospital & Research Centre, Riyadh, Saudi Arabia; b From the Department of Pathology & Laboratory Medicine, King Faisal Specialist Hospital & Research Centre, Riyadh, Saudi Arabia; c From the Department of King Faisal Cancer Centre, King Faisal Specialist Hospital & Research Centre, Riyadh, Saudi Arabia; d From the Department of Radiology, King Faisal Specialist Hospital & Research Centre, Riyadh, Saudi Arabia

## Abstract

Extramedullary adrenal plasmacytoma (EMP) involving the adrenal glands is rarely encountered clinicaly. We report a A 47-year-old male who presented with bilateral adrenal incidentalomas. After confirming EMP, the patient received two consecutive autologous hematopoietic stem cell transplants (HSCT) using high-dose melphalan. Following HSCT, a serial follow-up helical CT revealed a substantial decrease in the size of both adrenal masses. Serial periodic serum protein and urine electrophoresis and immunofixation showed abrogation of a previously noted monoclonal band. At 50 months follow-up the patient was alive and well. Our patient is the first with EMP to have received an autologous HSCT, which may prove to have a role in therapy due to the immunological effect of the infused donor marrow T-lymphocytes against the clonal proliferation of abnormal plasma cells in extrammedullary sites. This case indicates that an EMP should be added to the differential diagnosis of adrenal incidentalomas.

Plasmacytomas are tumors that arise in different locations due to the ubiquitous tissue distribution of plasma cells. They are a clonal proliferation of atypical plasma cells that exhibit a localized osseous or extraosseous (extramedullary) growth pattern.^1^ Extramedullary plasmacytomas (EMPs) have been described in a variety of locations.^1^ EMP involving the adrenal gland is unusual. In reporting the case, we wish to draw attention to its possibility in the differential diagnosis of adrenal incidentalomas.

## CASE

**Figure 1:**
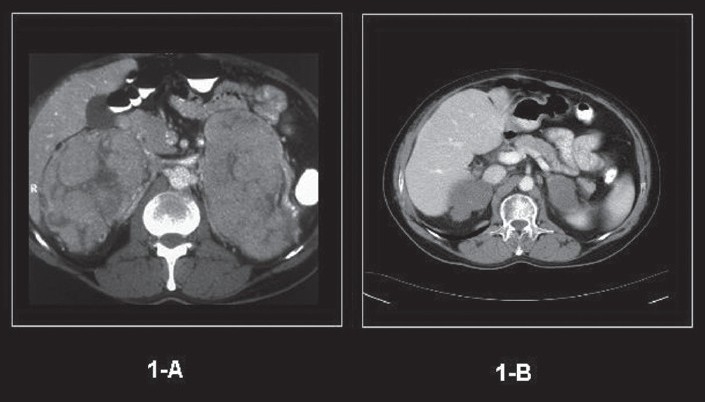
(A) CT scan abdomen, axial view following IV contrast administration (at presentation) revealed bilateral adrenal tumors with heterogeneous enhancement. The right adrenal tumor measured 11×8 cm while the left one measured 13×8 cm. Prominent vessels are demonstrated around the masses. Note the clear lobulations seperated by hypodense linear areas of probable fibrosis and necrosis within the tumor masses. (B) An axial contrast-enhanced CT image through the adrenals performed 47 months follow-up demonstrated marked interval decrease in both size and enhancement of bilateral adrenal masses. The right adrenal tumotr had decreased substantially to 5.2×4×5.7 cm and the left adrenal mass is regressed to 4.5×5×3.5 cm and showed findings of calcification and findings suggestive of fibrosis.

**Figure 2:**
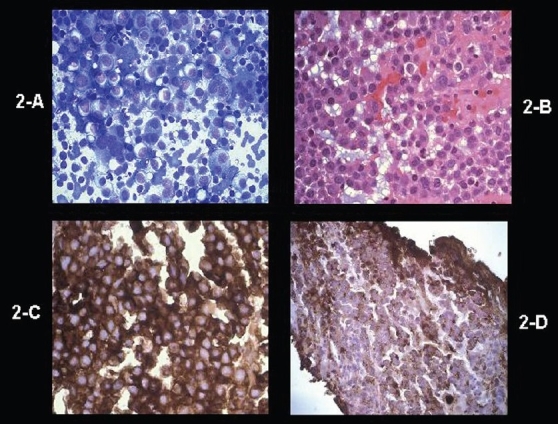
(A) Photomicrograph of the FNA biopsy specimen from right adrenal tumor showing clusters of plasma cells with eccentric nuclei, and prominent nucleoli; some of these cells show bi- and multi-nucleation (Diff Quick stain, ×400). (B) Photomicrograph of the biopsy specimen shows similar findings (hematoxylin and eosin stain, ×400). (C) Photomicrograph of the biopsy specimen from the adrenal tumor demonstrates plasma cells strongly positive for Kappa light chain (paraffin section, immunoperoxidase staining, ×400). (D) Photomicrograph of the biopsy specimen from the adrenal tumor shows strong staining of plasma cells with antibody to CD 38 (paraffin section, immunoperoxidase staining, ×400).

**Figure 3:**
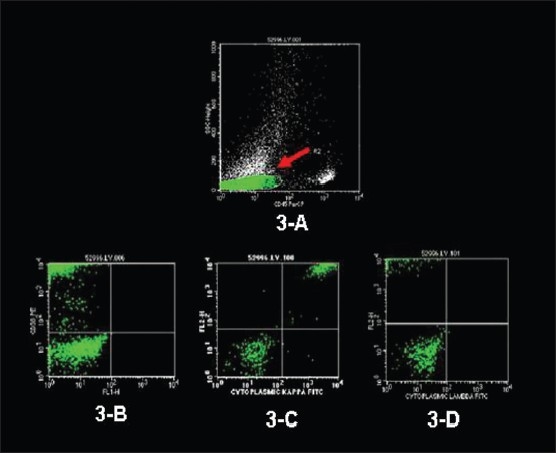
Flow cytometric histograms of adrenal plasmacytoma. Note the dim CD45 gate on upper panel (arrow) (A). Immunophenotyping revealed the monoclonal cytoplasmic kappa light chain expression in the CD38+ cells after permealization (3 bottom panels B-D).

**Figure 4:**
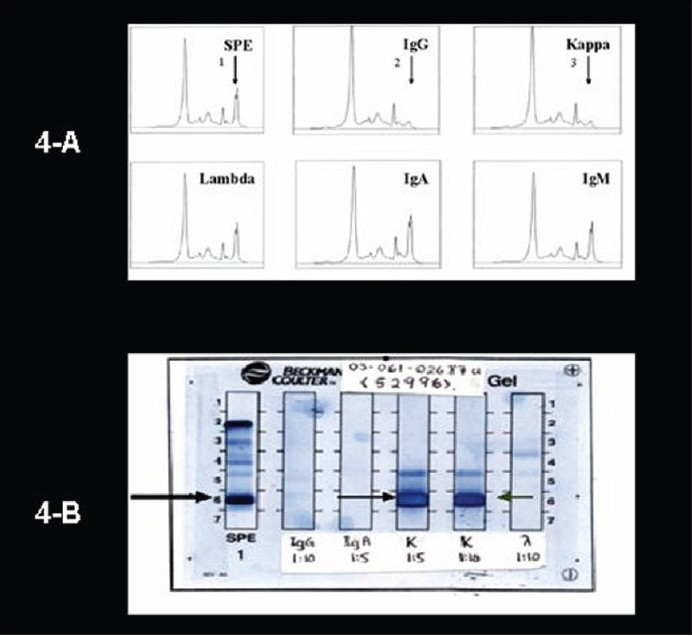
(A) Monoclonal protein typing by immunosubtraction on capillary zone electrophoresis (CZE). Note the distinct monoclonal protein peak (1) on CZE-SPE in the λ region, that is removed from the electrophoresis pattern by antisera to gamma (2) heavy chains (anti-IgG) and kappa light chains (3). The antisera to IgG lambda, IgA, and IgM had no effect (lower panel). The immunosubtraction indicated the monoclonal protein is IgG Kappa. (B) Immunofixation electrophoresis (IFE) of urine specimen for Bence-Jones protein (BJP) using Beckman Paragon system. Note the dense band of free kappa light chains corresponding to the band seen on urine protein electrophoresis (UPE). Note also the prozone effect (antigen excess) using 1:5 dilution, and its disappearance using 1:10 dilution.

A 47-year-old male presented at an outside hospital with painful right hip that was diagnosed as synovitis of the hip. A CT scan of the hip that included the abdomen detected bilateral adrenal masses. At presentation he was totally asymptomatic and other than large palpable bilateral abdominal flank masses had a totally negative physical examination. The patient underwent the following investigations: hemoglobin 149 g/L, red blood cells 5.37×10^12^/L, WBC 6.1×10^9^/L, serum creatinine 87 μmol/L (reference range, 65-129 μmol/L) serum calcium 1.95-2.25 g/L (reference range, 2.10-2.55 g/L), serum albumin 35 g/L (reference range, 42-48 g/L), serum phosphorus 0.57-1.27 mmol/L (reference range, 0.7-1.45 mmol/L), alkaline phosphatase 64-96 U/L (reference range, 40-115 U/L), total proteins 109 g/L (reference range, 65-81 g/L), and urine total protein of 1.22 g/L. The hormonal profile consisted of 24-hour urine normetanephrines of 2.88 μmol/day (reference range, 0-3.43 μmol), urine metanephrines of 0 μmol/day (reference range, 0-1.49), urine 3-methoxy-tyramine 1.34 μmol/day (reference range, 0-2.06), synthetic ACTH stimulation testing revealed a normal adrenal cortical reserve (base line serum cortisol, 301 nmol/L that increased to a peak of 564.7 nmol/L at one hour following intramuscular administration of 250 μg of 1-24 synthetic ACTH), urine cortisol of 45 μg/day (reference range, < 100 μg/day), urine aldosterone <33 nmol/day (reference range, 8-83 nmol/day), supine renin of 2.17 μg/L/h (reference range, 0.15-2.33 μg/L/h), and serum dehydroepiandrosterone sulfate of 0.7 μmol/L (reference range, 1.2-8.71). Imaging data consisted of an ultrasound of the abdomen/pelvis (not shown) that revealed large bilateral adrenal masses, a CT of the abdomen (Figure 1a without contrast and figure 1b following contrast administration) and an MRI abdomen (not shown) that showed massive bilateral adrenal masses, displacing the kidneys inferiorly, but that was otherwise negative. A skeletal survey (ribs, clavicle, scapulae, skull, spine, long bones of upper and lower extremities, pelvis), whole body bone scan and MRI spine showed no findings suggestive of multiple myeloma. Ultrasound-guided fine needle aspiration biopsy of the right adrenal mass (Figure 2a) and a core-needle biopsy (Figure 2b) showed morphological findings consistent with the diagnosis of plasmacytoma. Immunohistochemical staining data (Figures 2c and 2d) supported the diagnosis of plasmacytoma. Flow cytometry analysis of the adrenal tumor revealed an abnormal CD38+ cell population that exhibited monoclonal cytoplasmic kappa light chain expression, but negative for CD45, cytokeratin and lambda light chain (Figure 3). A bone marrow aspirate showed normocellular pattern with no evidence of multiple myeloma. Bone marrow flow cytometry for leukemia/lymphoma markers was negative. A distinct monoclonal protein band accounting for 44.6% of total serum proteins and 87.9% total gamma globulins was shown on serum protein electrophoresis. (Figure 4a). Immunofixation electrophorisis of urine specimen demonstrated free kappa light chain (Bence Jones proteins) (Figure 4b).

At initial treatment the patient received chemotherapy consisting of two cycles of vincristine, Adriamycin and dexamethasone (VAD) followed by two cycles of etoposide, dexamethasone, Ara-C and cisplatinum (EDAP). A follow-up CT scan showed no change in the size of the bilateral adrenal lesions nor was there a change in the immunological studies of serum and urine. Two years following initial presentation the patient received two consecutive autologous hematopoietic stem cell transplantation (HSCT) procedures using high dose melphalan. Starting 6 months pos-HSCT through a last follow-up at 50 months, serial periodic serum protein electrophoresis and immunofixation showed abrogation of a previously noted monoclonal band. Urine electrophoresis and urine immunofixation also turned out to be negative for Bence-Jones proteins. A last CT scan of the abdomen done at 47 month follow-up (Figure 1b) revealed that both adrenal tumors had regressed substantially and demonstrated findings of calcification and fibrosis. A follow-up skeletal survey at 47 months showed normal findings. At 50 months follow-up the patient was alive and well.

## DISCUSSION

The diagnosis in our case was based on the findings of a plasma cell tumor in an extramedullary site detected on adrenal imaging, the tissue diagnosis, the relevant immunological analysis of serum and urine, flow cytometry of the adrenal tumor, the absence of mutiple myeloma on bone marrow examination and the absence of skeletal lesions, coupled with the long-term follow-up data. An EMP is a rare malignant tumor. Fewer than 3% of all plasma cell proliferative disorders are EMPs.^2^ In the majority of instances (80% of cases), they occur in the upper respiratory tract or the oral cavity.^3^ The remainder arise in the gastrointestinal tract, central nervous system, breast, spleen, retroperitoneum, testes, and the thyroid or the lymph nodes.^3^

Extramedullary plasmacytoma involving the adrenal gland is unusual. It has been reported in 4 other cases previously. 3–6 Three of these cases were reported in middle-aged Japenese men presenting as adrenal incidentaloma who had an IgG monoclonal spike that was characterized as lambda light chain on immunoelectrophoresis. 3–5 In previously reported cases the adrenal plasmacytomas were all unilateral right-sided lesions and compared to our case were relatively smaller in size (3.5-4 cm). 3–6 All were hormonally non-functioning 3–6 as was our case and their follow-up data extended up to a year. Fujikata et al have reported no tumor recurrence after a year of follow-up following surgical excision and radiotherapy.^5^ Laproscopic adrenalectomy was done in 3 other cases reported by Kahara et al,^3^ Asahi et al,^4^ and Rogers et al.^6^

In contrast to previously reported cases, our case is dintinct in presenting with bilateral incidentalomas that were much larger, producing IgG kappa light chain, followed-up for a longer time period without surgical intervention but received chemotherapy that was considered a failure and had then undergone two consecutive autologous HSCT procedures. Following the first HSCT patient demonstrated a partial reduction of the adrenal masses and an improvement in protein electrophoresis. Considering these responses, a second HSCT procedure was undertaken that was temporally related to a significant reduction in the tumor size and resolution of the abnormal findings in serum and urine electrophoresis. These responses remained sustained without maintenance therapy. Because a biopsy was not performed from the contralateral left adrenal mass, we cannot definitely state that our patient indeed had bilateral plasmacytomas. However, the imaging findings, the follow-up data and the regression in the size similar to that of the right adrenal plasmacytoma following HSCT is highly suggestive of a bilateral process.

The role of HSCT in the management of EMPs is not defined. For the last 20 years autologus and allogeneic HSCT have been used to advantage in patients with multiple myeloma.^7^,^8^ However, there is no previous experience whatsoever regarding the use of HSCT in the management of adrenal plasmacytoma. Our patient with extramedullary plasmacytoma of the adrenal is the first to have received an autologous HSCT. HSCT may have a potential role, probably due to an immunological effect of the infused donor's marrow T-lymphocytes against the clonal proliferation of abnormal plasma cells in extrammedullary sites. We remain cautiously optimistic on the impact of consecutive autologous HSCT in EMP in our patient. Unquestionably, more experience is needed to study the effect of HSCT in extramedullary EMPs in general.

In conclusion, although the frequency of diagnosis of adrenal incidentaloma has increased with the increased use of contemporary imaging modalities, the definitive diagnosis of such lesions should rest on a combination of clinical and pertinent laboratory criteria coupled with cytological/histological criteria. Thorough investigations will lead to the diagnosis of most unusual adrenal lesions, which can have an enormous impact on treatment, quality of life and on eventual outcome. EMP should be considered among the differential diagnoses of adrenal incidentalomas.
